# A heat-flux upper boundary for modeling temperature of soils under an embankment in permafrost region

**DOI:** 10.1038/s41598-022-17529-w

**Published:** 2022-08-02

**Authors:** Tianyu Wang, Li-E. Yan

**Affiliations:** 1 College of Civil Engineering and Architecture, Guangxi University, 100 University Road, Nanning, 530004 Guangxi China; 2 The Key Laboratory of Disaster Prevention and Structural Safety of Ministry of Education, Guangxi University, Nanning, 530004 China

**Keywords:** Environmental sciences, Engineering

## Abstract

Building roads in permafrost region is challenged because permafrost is sensitive to temperature increase. As an embankment gains/drains heat mostly at the upper surface, accurately modeling the heat transfer in the upper surface is crucial to understand the thermal stability of the road. Popular methods treat the upper boundary as a temperature-controlled model (TCM), where temperature of the upper surface is set as a sinusoidal function. This simple function, however, fails to identify the influences of solar irradiance, heat convection, and thermal irradiance on the heat transfer on the ground surface. Here we introduce a heat-flux model (HFM) to calculate the heat fluxes at the embankment upper surface and at the adjacent ground surface. HFM-predicted temperature under an embankment is compared against the observed temperature to validate the model, and is compared to the TCM-predicted temperature. While TCM-predicted temperatures and HFM-predicted ones are similar in trend and in pattern, the HFM-predicted temperatures are far more coincident with the observed ones. The pros and cons of both HFM and TCM are discussed. Further studies are expected to use HFM to understand the heat flux components such as solar absorption, heat convection, and thermal irradiance on the temperature of permafrost under embankments.

## Introduction

Permafrost regions cover approximately 22.8 million km^2^ earth surface, of which permafrost in China makes up 1.59 million km^2^^1^. Roadways across permafrost regions are usually layered upon a built-up embankment to keep the underlying soil intact^2^. The construction of the embankment varies the permafrost stratum undesirably and causes differential settlement at the ground surface^3–6^. Accurately simulating the heat transfer at the ground surface is critical to precisely predict the temperature of the permafrost stratum after the construction of the embankment. When simulating the temperature of soils under a permafrost embankment, popular methods usually treat this boundary as a temperature-control model (TCM)^7–9^, in which the temperature at the ground surface is assumed a sinusoidal wave. This sinusoidal wave is used to bulkily represent the influences of solar radiation, heat convection, thermal irradiance, and evaporation (if any) on the ground surface^10^. While this model is simple, the parameters of the sinusoidal wave are estimated by rule of thumb, but not by art (Table 3) and is thus hard to be estimated accurately.

If the ground-surface temperature can be guessed reliably, the temperatures of the deeper soil vary less and thus can be estimated with greater confidence. Qin et al. has proposed a heat-flux upper boundary (HFUB) for the natural ground surface to predict the temperature of permafrost stratum^10,11^. The model is suitable to predict the temperature of permafrost under a flat, open area only. Liu et al. then proposed an earth-atmosphere coupled model to predict the temperature of soil under an embankment^12^. This model, however, does not consider the variation of daily solar irradiance. Without a HFM for predicting the temperature of the embankment and its underlying soils, it is unscientific to guide the selection of performance grading of asphalt binder for asphalt pavement under the specific climate condition; it is difficult to understand the partition of radiant heat flux at the ground and to find practical solutions that cut the heat gain of the embankment to preserve the underlying permafrost; it is unknown how to eliminate the sunny-shady effect of embankment in permafrost region by equalizing the conductive heat at both embankment side slopes. In addition, as the global warming heats up the underlying permafrost and causes deformation to the embankment, the HFM is indispensable to predict the settlement of the embankment precisely.

Here we propose a comprehensive heat-flux model (HFM) for the heat transfer at the embankment upper surface and at the natural ground adjacent to the embankment. The proposed model considers the heat flux from solar irradiance, heat convection, and thermal irradiance, and counters the shading effect of the embankment on the adjacent ground. Temperatures predicted by the HFM model are compared against the temperature profiles that are observed from a testing section at the Beiluhe Permafrost Station in the Qinghai-Tibet Plateau. The TCM is also used to predict the temperature at the same site to demonstrate the difference between the TCM and the HFM. The pros and cons of both the HFM and the TCM are explained. The application of the HFM is briefly discussed.

## Numerical models

The heat transfer in the embankment and in the soils below an embankment is conduction-dominated, which obeys

1 $$\rho {c}_{eq}\frac{\partial T}{\partial t}=\nabla \cdot (\lambda \nabla T)$$

where *ρ* (kg/m^3^) is the density of the soils; *T* (°C) is the temperature of the soils; *c*_*eq*_ (J/(kg K)) is the equilibrium heat capacity of the soils; and *λ* (W/(m⋅K)) is the heat conductivity of the soil. It is noted that *c*_eq_ represents the latent heat of the permafrost when the phase change of water in the permafrost occurs. Methods to calculate *c*_eq_ and *λ* have been documented well and can be referred in Appendix A of Supplementary Information.

**Figure 1:**
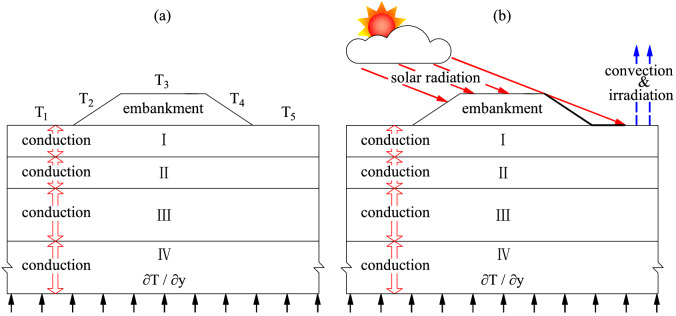
The computational domain of an embankment underlain by permafrost. (**a**) temperature-controlled upper boundary, (**b**) heat-flux upper boundary. *T*_*i*_ (*i* = 1, 2, …, 5) stands for a specific sinusoidal temperature function.

To solve Eq. (1), one must specify a proper boundary condition to the computational domain, which should be sufficiently large so that a further extent of the domain does not influence the temperature simulation of the embankment and of the underlying soils. As an embankment is usually an isosceles trapezoid with the short base at the top, the computational domain is selected as the trapezoid centered over a rectangle envelop that represents the natural ground (Fig. 1). The heat flux from right and left sides of the rectangle envelop is adiabatic, and the heat flux from the bottom of the base is the product of the local geothermal gradient and the heat conductivity of the soil at the base (Fig. 1). Most of heat fluxes that change the temperature of the embankment and of the underlying soils come from the upper boundary, which consist of the embankment upper surface, the side slope surface, and the adjacent natural ground surface (Fig. 1). In published articles^10,13^, the upper boundary can be treated as either a temperature-controlled boundary (Fig. 1a) or a heat-flux boundary (Fig. 1b).

### Temperature-controlled model (TCM)

Popular models to predict the temperature of the permafrost under a roadway embankment prefer using a temperature boundary, which is termed TCM. The TCM specifies to the upper boundary as a sinusoidal temperature function

2 $${T}_{s}={T}_{0}+A\mathrm{cos}(\varpi t+\phi )$$

where *T*_*s*_ (°C) is the ground-surface temperature, *T*_0_ (°C) represents the mean annual ground temperature, and *A* (°C) is the amplitude of the ground-surface temperature, *ω* (rad/s) represents the angular frequency, *ω* = 2π/(24 × 3600 × 365); and *ϕ* stands for the phases (in radians) of annual ground surface temperature.

Equation (2) considers the daily temperature only, although some models occasionally take the daily temperature pattern into account^14^. As the ground-surface temperature is difficult to be measured directly, *T*_0_ and *A* are coarsely estimated from the annual air temperature (data from the nearly weather station) according to the adhesive layer theory^15^. Parameters in Eq. (2) bulkily represent the complex heat transfer at the ground surface, without considering the solar irradiance, convection, thermal irradiance, and evaporation.

### Heat-flux model (HFM)

#### Heat flux at boundary of the computational domain

The HFM focuses on formulating the upper boundary to solve the temperature of soils in the computational domain using Eq. (1). The most variable heat-flux boundaries are the ground surfaces and the embankment’s upper surface. Both surfaces absorb irradiance from the sun and downward thermal irradiances from the outer space *L*_*d*_ (W/m^2^). Both surfaces release a portion of the absorption as heat convection *H* (W/m^2^), upward long-wave radiation to the outer space *L*_*u*_ (W/m^2^), and evaporation *E* (W/m^2^) (if any) simultaneously. The surplus heat flux, *G* (W/m^2^), stores at the ground surface to vary the skin temperature and conducts it to the deeper soils. The heat balance of the upper surface exposed to the air obeys,

3 $$I\left(1-R\right)+{L}_{d}=G+H+{L}_{u}+E$$

where *I* (W/m^2^) is the incoming solar irradiance to the surface, *R* (–) is the reflectance (albedo) of the surface.

In Eq. (3), *L*_*u*_ and *L*_*d*_ are proportional to the ground-surface thermal emissivity, *ε* (–); in practice, it is more convenient to compute the net long-wave radiation, which is

4 $$L={L}_{u}-{L}_{d}=\varepsilon \sigma \left({\left({T}_{s}+273.15\right)}^{4}-{\left({T}_{y}+273.15\right)}^{4}\right)$$

where *σ* is the Stefan-Boltzmann constant, *σ* = 5.67 × 10^−8^ W ·m^−2^ ·K^−4^; the subscripts “*s*” and “*y*” represent the ground surface and the outer sky, respectively.

In Eq. (4), the ground-surface temperature, *T*_*s*_, can be calculated from Eq. (1) once the heat flux is specified to the boundary of the computational domain. The sky temperature, *T*_*y*_ (°C), can be estimated:

5 $${T}_{y}={\varepsilon }_{y}^{0.25}{(T}_{a}+273.15)-273.15$$

where *T*_*a*_ (°C) is the air temperature; *ε*_*y*_ (–) is the sky emissivity, which is

6 $${\varepsilon }_{y}=0.754+0.0044{T}_{d}$$

where *T*_*d*_ (°C) is dew point:

7 $${T}_{d}={b}_{0}{r}_{0}/({a}_{0}-{r}_{0})$$

where *a*_0_ = 17.3, *b*_0_ = 237.7, and *r*_0_ = *a*_0_*T*_a_/(*b*_0_ + *T*_*a*_) + ln(*χ*/100)^12^, with *χ* is the relative humidity of the surrounding air.

The heat convection, *H*, can be calculated by

8 $$H={h}_{c}({T}_{s}-{T}_{a})$$

where *h*_*c*_ (W/(m^2^·°C)) is the heat convection coefficient. The computation of *h*_*c*_ has been well documented in many models, among which the following simple one can provide reliable accuracy^16^

9 $${h}_{c}=\left\{\begin{array}{c}5.6\times 4.0v v<5\\ 7.2\times {v}^{0.78} v\ge 5\end{array}\right.$$

where *v* (m/s) is the wind speed recorded at a height of 9.0 m. For a wind recorded from a weather station that is not set at 9.0 m height, the wind speed must be converted according to

10 $$v={{v}_{z}\left(\frac{z}{9}\right)}^\frac{1}{7}$$

In Eq. (3), the evaporation *E* is difficult to calculate because it is hard to know both the water availability at the ground surface and the evaporation resistance of the ground surface. When water is available at the ground surface, the evaporation is proportional to the incoming solar radiation, with the peak evaporation occuring around solar noon and with a negligible evaporation at nighttime. According to Qin et al.^10^, the evaporation term can be reasonably neglected by calibrating the solar reflectance at the ground surface. In this model, the evaporation is neglected, and the ground-surface reflectance is adjusted unless the observed ground temperature is coincident with the predicted one. As a result, the heat flux to the upper boundary of the computational domain is

11 $$G=I\left(1-R\right)-H-L$$

#### Sunlit and shade of the side slope of an embankment

In Eq. (11), formulas to calculate the components *H* and *L* have been shown. The most complex component in Eq. (11) is the solar irradiance, *I*, on the embankment surface and on the ground surface adjacent to the embankment.

Solar irradiance on a global horizontal surface is the global horizontal solar irradiance logged from the local weather station. Solar irradiance is partitioned to beam radiation and diffuse radiation. The side slope of an embankment can be either shaded or sunlit, depending on the time of the day, on the day number of the year, on the orientation of the embankment, and on the angle of the side slope. The solar irradiance on the side slope is thus more complicate. To find the solar irradiance on a tilt surface, one must find the solar azimuth angle, which varies with day and time. On a specific day, the solar declination angle, *δ* (rad), can be estimated from

12 $$\delta =0.409\mathrm{sin}(2\uppi \frac{N+284}{365})$$

where *N* is the day number of the year, with *N* = 1 on January 1.

On a specific time of the day, the solar zenith angle can be estimated from

13 $$\mathrm{cos}\theta =\mathrm{sin}\delta \mathrm{sin}\beta +\mathrm{cos}\delta \mathrm{cos}\beta \mathrm{cos}\alpha$$

where *β* (rad) is the latitude of the observer; *α* (rad) is the solar hour angle, which is 0 at solar noon. The time span from sunrise to sunset can be derived from:

14 $$\mathrm{cos}\alpha =\frac{\mathrm{sin}(-0.83^\circ )-\mathrm{sin}\delta \mathrm{sin}\beta }{\mathrm{cos}\beta \mathrm{cos\delta }}$$

where *α* at the sunset time is positive, and at the sunrise time is negative.

With the solar declination angle, the solar zenith angle and the solar hour angle, one can find the solar azimuth angle, $\gamma$ (rad), is given by

15 $$\mathrm{sin }\gamma =\mathrm{cos}\delta \mathrm{sin}\alpha /\mathrm{sin}\theta$$

If $\left|\mathrm{sin }\gamma \right|>1$ or if $\left|\mathrm{sin}\theta \right|$ in Eq. (15) is infinitesimal, the solar azimuth angle has to be estimated by:

16 $$\mathrm{cos}\gamma =\frac{\mathrm{cos}\theta \mathrm{sin}\beta -\mathrm{sin}\delta }{\mathrm{sin}\theta \mathrm{cos}\beta }$$

It is noted that at noon *θ* = 0, the denominator in Eqs. (15) and (16) is equal to zero. To circumvent this problem, one can discrete the time sequence between sunrise to sunset in an even number so that the sequence does not have *θ* = 0.

With the solar azimuth angle, one can determine if a side slope of an embankment is sunlit or shaded. At low-sun case, beam radiation hits one side slope only, while the other is shaded. Assuming the height of the embankment is *h* (m), beam radiation creates a shaded belt parallel with the embankment orientation. The width of the belt is

17 $${x}_{0}=h\mathrm{tan}\theta \mathrm{cos}(\gamma -{\gamma }_{e})$$

where *γ*_*e*_ (rad) is the orientation of the embankment.

Whether a side slope of an embankment is sunlit or shaded can be estimated by

18 $$\left\{\begin{array}{ll}{x}_{0}\ge h\mathrm{cot}(\eta )& \mathrm{south}/\mathrm{east}-\mathrm{facing side slope is shaded}\\ {x}_{0}\le -h\mathrm{cot}(\eta )& \mathrm{north}/\mathrm{west}-\mathrm{facing side slope is shaded}\\ -h\mathrm{cot}(\eta )<{x}_{0}<h\mathrm{cot}(\eta )& \mathrm{both side slopes is sunlit}\end{array}\right.$$

where *η* (rad) is the angle of the side slope of the embankment.

#### Solar irradiance on the upper surface of the computational domain

Global solar horizontal solar irradiance, *I* (W/m^2^), is divided into beam irradiance *I*_*b*_ (W/m^2^) and diffuse irradiance *I*_*d*_ (W/m^2^), that is

19 $$I={I}_{d}+{I}_{b}$$

The division has been documented in many models, in which one simple, well-cited model can provide reasonable accuracy^17^:

20 $$\frac{{I}_{d}}{I}=\left\{\begin{array}{l}1.0-0.249{k}_{y} , {k}_{y}<0.35 \\ 1.557-1.84{k}_{y}, 0.35\le {k}_{y}\le 0.75\\ 0.177 {k}_{y}>0.75\end{array}\right.$$

where *k*_*y*_ is the sky cleanness factor with 0 representing a fully cloudy day and 1.0, a fully sunny day.

The global horizontal solar irradiance, *I*, can be directly specified to the upper surface of the embankment. However, the solar irradiance on the side slope and on the natural ground surface adjacent to the embankment is more complicate. According to Eq. (18), if a surface is sunlit, the solar irradiance is *I*_*d*_ + *I*_*b*_ falling on that surface; otherwise, is the *I*_*d*_ only. Diffuse irradiance on the side slope is

21 $${I}_{ds}={I}_{d}(1+\mathrm{cos}\eta )/2$$

The beam radiation on the side slope is the dot product of the beam irradiance *I*_*b*_ and the normal of the side slope.

22 $${I}_{bs}={I}_{b}(\mathrm{sin}\theta \mathrm{sin}\eta \mathrm{cos}\left(\psi - \gamma \right)+cos\theta \mathrm{cos}\eta )$$

where the azimuth of the side slope, *ψ* (rad), can be estimated by

23 $$\begin{gathered} \psi = {\uppi } - \gamma_{e} ,{\text{ face south}}/{\text{east}} \hfill \\ \psi = 2{\uppi } - \gamma_{e} ,\,{\text{face north/west}}\, \hfill \\ \end{gathered}$$

In Eq. (22), if *I*_*bs*_ < 0, which means the slope is shaded, then *I*_*bs*_ = 0. The solar irradiance on the side slope is

24 $${I}_{s}={I}_{ds}+{I}_{bs}$$

The solar irradiance on the natural ground surface, *I*_*g*_, is also the addition of diffuse irradiance *I*_*dg*_ and beam irradiance *I*_*b*g_. The construction of the embankment blocks the sky view of the natural ground surface, especially the surface that is close to the side slope toe of the embankment. Setting the side slope toe is the origin, the x axis perpendicular to the embankment orientation, and the y axis normal to the ground surface, one gets the sky view factor of any point (*x*,0) at the natural ground surface as

25 $${F}_{x\to \mathrm{sky}}=0.5+0.5\frac{h\mathrm{cot}\left(\eta \right)+x}{\sqrt{{h}^{2}+{\left(h\mathrm{cot}\left(\eta \right)+x\right)}^{2}}}$$

The diffuse radiation on the natural ground surface adjacent to the side slope is

26 $${I}_{dg}={F}_{x\to \mathrm{sky}}{I}_{d}$$

The beam radiation on the natural ground surface is

27 $${I}_{bg}=\left\{\begin{array}{cc}{I}_{b}\mathrm{cos}\theta & \mathrm{sunlit}\\ 0& \mathrm{shaded}\end{array}\right.$$

The solar irradiance on the natural ground surface adjacent to the side slope is

28 $${I}_{g}={I}_{dg}+{I}_{bg}$$

It is noted that a factor of sunlight reflecting from the side slope can be captured by the adjacent natural ground surface, and vice versa. This solar trapping effect increases the solar absorption of the side slope and of the adjacent ground, especially for the surface near the side slope toe. This topic has been documented and it is found the additional solar absorption caused by the solar trapping effect is negligibly small compared to the solar absorption^18^.

### Simulations information

This study uses both the TCM and HFM to simulate the temperature of underlying permafrost under an embankment in the Beiluhe Permafrost Station (34.8° N, 92.9° E). The simulated results are compared against the logged temperature. The embankment is isosceles with a height of 3 m, a top (short) base of 8.0 m, and a lower (long) base of 17 m. The computational domain is extended horizontally 20 m from the side-slope toe of the embankment, and vertically 22 m from the natural ground surface. The orientation of the embankment is set as east–west orientation, because the thermal asymmetry of this embankment is the most-deleterious case. The embankment filler consists of coarse grained soil. The computational domain in the natural ground, from top to down, consists of a 2 m-thick loose silty clay, a 2 m-thick silty clay, a 6 m-thick layer with ground ice and clay, and finally a 12 m-thick mudstone silty clay (Fig. 1). Soils’ thermal parameters used for the simulation are listed in Table 1. Both left and right sides of the computational domain are set as adiabatic, and the thermal gradient at the bottom of the domain is 0.03 °C/m.

**Table 1:** Thermal parameters of the soils in the Beiluhe Permafrost Station.

Layer	*ρ* (kg/m^3^)	*w* (%)	*a*	*b*	*k* (W m^-1^ K^-1^)		*c* (J kg^-1^ K^-1^)	
					*k*_*u*_	*k*_*f*_	*c*_*u*_	*c*_*f*_
Embankment	2111	8	0.053	0.209	1.433	1.537	1050.5	892.6
I	2227	8	0.021	0.310	1.485	1.525	866.3	856.3
II	2130	12	0.052	0.520	1.449	1.528	991.4	870.8
III	1980	40	0.112	0.519	1.242	1.642	1766.6	1157.3
IV	2180	8	0.053	0.281	1.433	1.537	1050.2	892.9
Water	1000	–	–	–	0.600	2.000	2050.0	4181.3

Data can be referred from paper^10^; physical meanings of some parameters are referred to the Appendix A of Supplementary Information.

Firstly, the heat-flux boundary is specified to the upper surface of the computational domain. The air temperature *T*_*a*_, the global horizontal solar radiation *I*, and the wind speed *v*, the relative humidity *χ*, and the sky cleanness factor *k*_*y*_ at the embankment site are logged from typical annual weather data. The solar irradiance on the top of the embankment is Eq. (19); that on the side slope, Eq. (24); and that on the adjacent ground surface, Eq. (28). The solar reflectance of the embankment top, side slope, and adjacent ground is set as 0.22 according to Qin et al.^19^. Considering that water evaporates when it is available at the topsoil and that the evaporation pattern is coincident with solar radiation pattern, the simulation adjusts the reflectance some degree unless the predicted temperatures are best matched with the observed ones.

Then, the TCM is used to simulate the permafrost temperature at the same site. As a guess of the parameters (*T*_0_, *A*, and *ϕ*) in Eq. (2) could cause substantial errors, this study does not use the adhesive layer theory to parameterize the average, amplitude, and phase of annual ground-surface temperature. Instead, this study uses the least square fitting method to fit the ground-surface temperatures that are predicted via the HFM. The fitting *T*_0_, *A*, and *ϕ* in Eq. (2) are used as the parameters for the TCM to simulate the soil temperature under the embankment, while the soils’ thermal properties are kept unchanged.

In numerical simulation, either the HFM or the TCM needs specifying an initial temperature to the embankment and to the soil under the embankment. Initial temperatures of the natural ground soils are obtained by repeatedly using the heat-flux boundary to simulate the ground temperature (without embankment) until the differences of the temperature profile at a specific time of two sequential years are less year 0.01 °C. The temperature profile at 00:00 (hh:mm) on September 30 is treated as the initial temperature because the construction of the embankment is completed around this date usually. The initial temperature of the embankment is set as 2 °C uniformly, as usually used in published articles on this topic. This initial temperature (for the soil and the embankment) is used to simulate the temperature of the soils for 50 years after the construction of the embankment.

The soil temperature predicted by the HFM is compared against the temperature logged an earthen embankment in the Beiluhe Permafrost Station from September 2001 to October 2006. The embankment, DK1136 + 400, is an earthen roadbed of the Qinghai-Tibet Railway. Thermistors were buried inside the embankment central borehole and inside the boreholes rightly below the side slope toes to log the soil temperature. In addition, at the natural ground that was 20 m away from the embankment side slope toe, a borehole was also drilled to log temperature at the natural ground. For convenience, the temperature reported in the following section are the daily mean temperature, unless otherwise noted.

### Validation method

Since it was assumed that the ground thermal regime has reached an equilibrium state before the construction of the roadbed. Therefore, to ascertain the ground temperature error caused by the iteration, field-observed temperature from September 7, 2001 to September 7, 2006 was compared with that of simulation. In addition, we adopted the coefficient of determination (R^2^), mean value (Mean), and standard deviation (Std) to examine the accuracy of the simulation results, where all three indicators were calculated using MATLAB software. The specific calculation methods are as follows:

29 $${R}^{2}=1-{\sum_{i=1}^{n}\left({T}_{o,i}-{T}_{s,i}\right)}^{2}/{\sum_{i=1}^{n}\left({T}_{o,i}-\overline{{T }_{o}}\right)}^{2}$$

30 $$\mathrm{Mean}=\sum_{i=1}^{n}\left({T}_{o,i}-{T}_{s,i}\right)/n$$

31 $$\mathrm{Std}=\sqrt{\frac{1}{n}\sum_{i=1}^{n}{\left[\left({T}_{o,i}-{T}_{s,i}\right)-\sum_{i=1}^{n}\left({T}_{o,i}-{T}_{s,i}\right)/n\right]}^{2}}$$

where *T*_o_, *T*_s_, $\overline{{T }_{o}}$ are the observed temperature, the simulated temperature and the average value of the observed temperature, respectively; *n* represents the total number of values (n = 1, 2, 3, …), and the subscript *i* stands for the serial number of the values.

## Results

### Validation of the HFM

**Figure 2:**
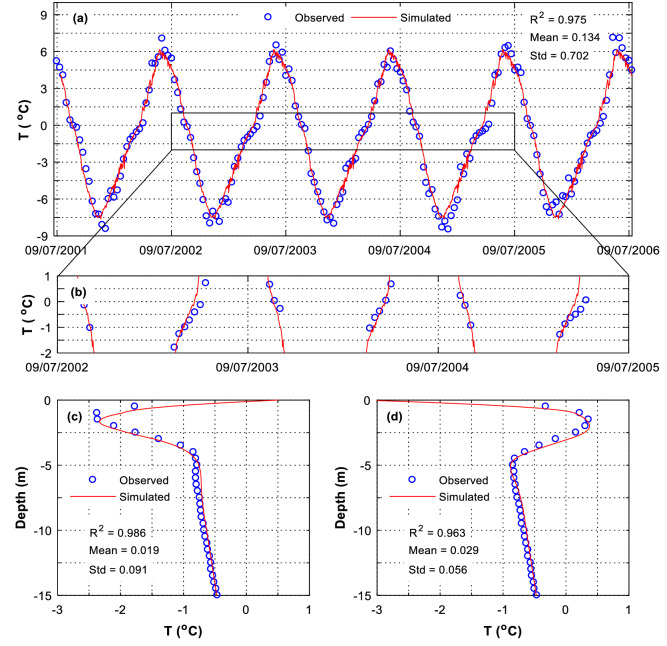
Soil temperature predicted by HFM. Location: at the natural borehole that is 20 m away from the side-slope toe (**a**) temperature serial at 0.5 m depth, with R^2^ = 0.975, Mean = 0.134 °C and Std = 0.702 °C, (**b**) temperature serial at 0.5 m depth, (**c**) on April 20, 2002, and (**d**) on October 19, 2002.

Figure 2a,b show the temperature of the soils at 0.5 m depth under the natural ground that is 20 m away from the embankment side slope. It is found that the predicted temperatures (straight line) are well coincident with the simulated ones (dotted data), with R^2^ = 0.975, a mean error of 0.134 °C and a standard deviation of 0.702 °C (Fig. 2a,b). This statistical data substantiates that the HFM can well predict the temperature of the permafrost under the embankment. The good agreement between the simulation and the observation can be further substantiated from the temperature profiles in the natural borehole. As shown in Fig. 2c, the predicted temperature profile on April 20, 2002 is well agreed with the observed one. Similarly, in Fig. 2d, the predicted temperature profile is highly coincident with the observed one, with R^2^ = 0.963, a mean error of 0.029 °C and a standard deviation of 0.056 °C.

**Figure 3:**
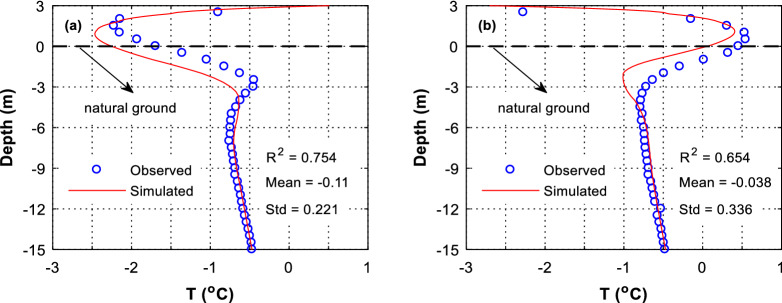
Soil temperature predicted by HFM. Location: at the central borehole. (**a**) on April 20, 2002, and (**b**) on October 19, 2002.

We further compare the predicted temperature profiles at the central borehole of the embankment to the observed ones. It is found that the predicted temperature near the embankment upper surface is greatly deviated from the observed one (Fig. 3). This great deviation is because the embankment is assumed to be completed as September 30 and the embankment temperature is assumed as 2 °C uniformly. These assumptions are somewhat unrealistic because the real construction of the embankment takes months and the temperature of the earthen filler is unknown. Despite this great deviation at near the ground surface, the predicted temperatures at deeper layer are well agreed with the observed ones (Fig. 3). As the heat of the embankment drains overtime and the influence of the initial temperature on the simulation vanishes, it is believed that the HFM can well predict the temperature of the soil under the embankment.

### Comparison of the HFM against the TCM

Here we compare the soil temperatures predicted by the TCM against the observed ones. To impose the TCM to the embankment surfaces, one must know *T*_0_, *A*, and *ϕ* in Eq. (2). In this study, the least square fitting method is used to fit the HFM-predicted ground-surface temperature to get *T*_0_, *A*, and *ϕ* in Eq. (2). The values of *T*_0_, *A*, and *ϕ* tabulated in Table 2 are used, while the others are the same as those used in the HFM.

**Table 2:** A serial of guess values for *T*_0_ and *A*, *ϕ*.

Location	*T*_0_ (°C)	*A*	*ϕ*	R^2^
Natural ground surface	− 0.76	8.04	1.03π	0.97
Upper surface	− 0.76	8.16	1.03π	0.97
Southern side slope	− 0.60	6.53	1.09π	0.93
Northern side slope	− 2.40	7.58	1.08π	0.96

**Figure 4:**
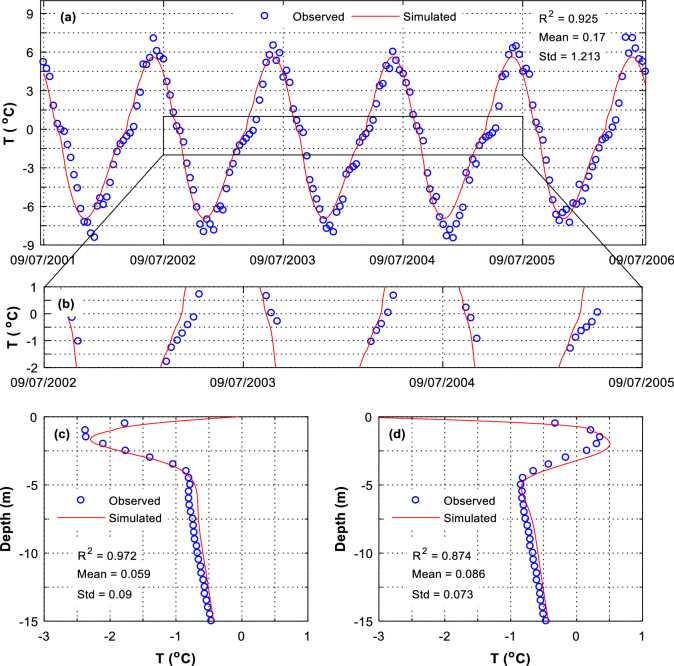
Soil temperatures predicted by TCM. Location: at the natural borehole that is 20 m away from the side-slope toe. (**a**) Temperature serial at 0.5 m depth, with R^2^ = 0.925, Mean = 0.17 °C and Std = 1.213 °C, (**b**) temperature serial at 0.5 m depth, (**c**) on April 20, 2002, and (d) on October 19, 2002.

**Figure 5:**
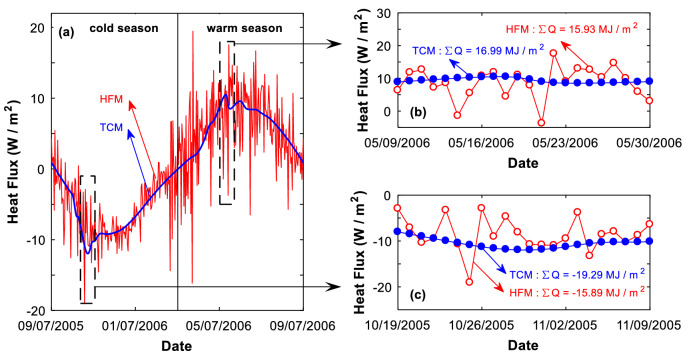
During the freezing/thawing period, the TCM imposes more energy to the natural ground surface than the HFM does. (**a**) The entire year, (**b**) the thawing period, and (**c**) the freezing period.

It is found that the TCM can also predict the temperature of the soil under the natural ground. As shown in Fig. 4a,b, the temperature serial at 0.5 m depth is well coincident with the observed one, with a R square of 0.925, a mean error of 0.17, and a standard deviation of 1.213 °C. A close comparison between the predicted and observed temperatures reveals that when the temperature is close to the freezing/ thawing point (− 1 to 0 °C), the predicted temperature serial increases or decreases faster than the observed one (Fig. 4b). In reality, when the temperature of a soil is close to the freezing/thawing point, the soil needs more heat (cold) to increase (decrease) its temperature (Fig. 5a). But the TCM unrealistically supplies the sufficient heat (cold) to the soil such that the temperature at the ground surface follows a sinusoidal function (Fig. 5b,c). As a result, the TCM-predicted temperatures deviate somewhat from the observed ones, especially for temperature of the soils at shallow ground where they weather cyclic thawing and freezing annually (Fig. 4c,d). At deeper ground where the soil remains frozen, the predicted temperatures are well agreed with the predicted ones (Fig. 4c,d).

**Figure 6:**
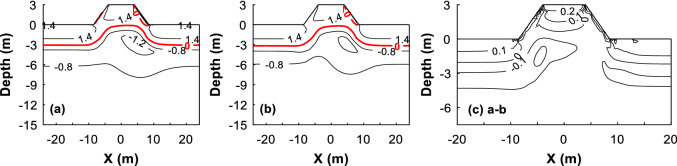
Comparison between the HFM-predicted temperatures and TCM-predicted ones after the embankment is constructed for 5 years. Date: On October 19. (**a**) HFM-predicted temperature, (**b**) TCM-predicted temperature, and (**c**) the HFM-predicted temperatures to minus the TCM-predicted ones.

As the temperature profiles and temperature serial in the natural boreholes and central borehole cannot represent the temperature of the entire embankment. Here we show the embankment’s temperature contours that are predicted by the HFM and the TCM, respectively. In case that the embankment has been built for five years, the daily mean temperature predicted by the HFM, in pattern and in magnitude, are highly similar with those predicted by the TCM (Fig. 6a,b). Using the HFM-predicted temperatures to minus the TCM-predicted ones, one can find the difference between the two modelings is about 0.1–0.2 °C (Fig. 6c), which is negligibly small and further implies that both models can predict the temperature of the soil under the embankment. Similar differences and similar patterns are found when the predicted embankment’s temperature contours in 10th year and 50th year are compared (Appendix B in Supplementary Information).

**Figure 7:**
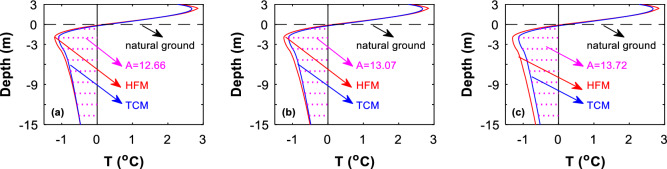
The central-borehole temperature profile predicted by HFM is compared with that by TCM. Date: on October 27. (**a**) 5th year, (**b**) 10th year, and (**c**) 50th year.

**Figure 8:**
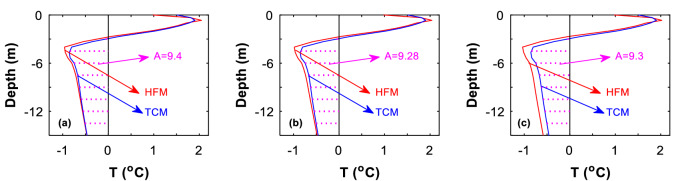
The southern side-slope borehole temperature profile predicted by HFM is compared with that by TCM. Date: on October 27. (**a**) 5th year, (**b**) 10th year, and (**c**) 50th year.

**Figure 9:**
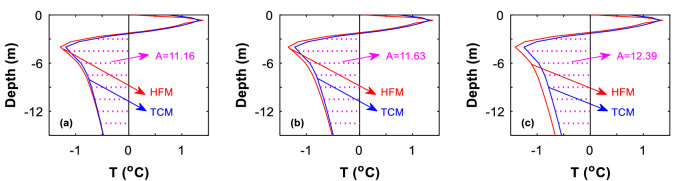
The northern side-slope borehole temperature profile predicted by HFM is compared with that by TCM. Date: on October 27. (**a**) 5th year, (**b**) 10th year, and (c**)** 50th year.

To further understand the difference between the two models, we analyze the temperature profiles at the embankment central borehole and at the southern and northern side-slope toes. It is found that at the central borehole, the temperature profile predicted by the HFM is closely matched with that predicted by the TCM, especially within 5 year after the construction of the embankment (Fig. 7a). Almost the same difference is observed when compared is the temperature profiled predicted by the two models at 10 year after the construction of the embankment (Fig. 7b). As time elapses, the TCM-predicted temperature is about 0.1–0.2 °C higher than the HFM-predicted model, especially at the deeper layers (Fig. 7c). Similar differences and similar deviations between the two models are observed at the temperatures under the side slopes toes (Figs. 8 and 9). We do not know the reason why the TCM-predicted temperature is about 0.1–0.2 °C higher than the HFM-predicted model, a phenomenon that deserves further investigation.

## Discussion

### The difference between HFM and TCM

This study is not intended to referee that the HFM is superior to the TCM, or vice versa. Each model has its advantage and disadvantage. The HFM mimics the natural heat transfer at the ground surface and thus can predict the temperature of the soil under the embankment more precisely. The HFM includes the influences of embankment thermal properties and weather data on the temperature of a permafrost stratum. It is thus capable to use the HFM to perform a sensitivity study about the influence of each parameter on the soil temperature. The HFM can help identify if variation of a specific parameter, such as solar reflectance, is favorite to preserve the permafrost under the embankment. If the deformation of the permafrost soil is concerned, it is preferred to use the HFM model to precisely predict the permafrost temperature so that the temperature-related deformation of the soil is estimated properly. In comparison, the TCM model is not a realistic boundary because the ground-surface temperature is not a rigidly sinusoidal wave. The TCM-predicted temperatures could deviate more from the observed ones. But the TCM can predict the temperature of the permafrost in a pattern that is similar to the observed one. As a result, if the trend of the temperature of permafrost under an embankment is interesting, the TCM is superior to the HFM because it is simple and it costs very little simulation time.

Currently, simulation of the temperature of permafrost soils under an embankment needs to guess some parameters. The TCM needs to reasonably guess the mean annual ground-surface temperature, *T*_0_, and the amplitude of this temperature, *A*. In this study, *T*_0_, *A*, and *ϕ* are regressed from the HFM-predicted temperature. As a result, the TCM-predicted temperature is coincident with the observed ones. However, in most cases, *T*_0_, *A*, and *ϕ* are unknown. Many documented articles states that *T*_0_, *A*, and *ϕ* are estimated on the basis of the adhesive layer theory, in which both *T*_0_ and *A* are some cesium degree greater than the local air temperature. But how to estimate both *T*_0_ and *A* is seldom explained. Table 3 lists *T*_0_, *A*, and *ϕ* that have been used to predict the temperature of permafrost under embankments in the Qinghai-Tibet Plateau. It can be seen that the *T*_0_, *A*, and *ϕ* vary greatly and that their values, sometime, are specified empirically and randomly.

**Table 3:** A list of *T*_0_ and *A* which have been used in simulated temperature of permafrost.

References	Parameters			∂*T*/∂*t* (°C/year)	Surfaces	Studies area
	*T*_0_ (°C)	*A* (°C)	*Φ* (rad)			
^13^	− 1.5	12	π/2	0.05	NG	An altitude of 4500 m in the QTP
	0.7	13	π/2	0.05	S	
	1.5	15	π/2	0.05	Ballast pavement	
^20^	− 0.4	12	π/2	–	NG	An altitude of over 4500 m in the QTP
	1.8	15	π/2	–	S	
	2.6	15	π/2	–	Ballast pavement	
^21^	− 1.0	12	7π/12	–	NG	An altitude of 4500 m in the QTR
	2.1	13	7π/12	–	SS	
	0.3	13	7π/12	–	NS	
	2.0	15	7π/12	–	Ballast pavement	
^22^	2.3	12.0	π/2	0.05	NG	An altitude of 4500 m along the QTR
	3.3	13.0	π/2	0.05	S	
	4.5	14.0	π/2	0.05	Ballast pavement	
^23^	− 0.5	11.5	π/2	0.02	NG	The Beiluhe basin along the QTR
	1.0	14.5	π/2	0.02	S	
	1.0	14.5	π/2	0.02	Gravel pavement	
^24^	0.4	12.0	π/2	0.05	NG	The Beiluhe basin along the QTP
	− 0.1	14.2	π/2	0.05	S	
	1.0	15.2	π/2	0.05	Ballast pavement	
^25^	− 1.3	12.0	–	0.05	NG	The Beiluhe basin along the QTP
	1.4	13.2	–	0.05	SS	
	− 1.2	15	–	0.05	NS	
	0.4	14.5	–	0.05	Gravel pavement	
^26^	− 0.8	11.5	π/2	0.02	NG	The Beiluhe basin along the QTP
	− 0.7	6.5	π/2	0.02	SS	
	− 1.6	7.5	π/2	0.02	NS	
	0.6	11.0	π/2	0.02	Gravel pavement	
^27^	− 1.0	11.5	π/2	0.04	NG	South of the Beiluhe Basin on the QTP
	4.1	10.6	π/2	0.04	SS	
	0.1	12.0	π/2	0.04	NS	
	5.3	15.6	π/2	0.04	Asphalt pavement	

“–” do not be measured, *NG* natural ground surface, *S* side slope, *SS* south-facing side slope, *NS* north-facing side slope, *QTR* Qinghai-Tibet Railway, *QTP* Qinghai-Tibet Plateau.

While the HFM does not need *T*_0_, *A*, and *ϕ*, it needs to guess some parameters like sky clearness factor, solar reflectance and others. As the heat in the ground comes primarily from the sunlight, the most challenge of the HFM is to guess a reliable solar reflectance *R* on the upper surface. Although the *R* value of a surface can be measured, the true *R* value varies during the courses of day and year, with the lowest reflectance in summer and greatest value in winter^12^. It has been advocated that the use of energy-based *R* value to replace the true *R* so that the seasonal and daily variations of the solar reflectance at the embankment surface are weighed. The data for the energy-based *R* value, however, is very limited currently^28^. Specifying a reliable *R* value for the upper ground surface is thus challenged. Further studies are thus expected to understand the solar reflectance of ground surface in permafrost regions.

### The application of the HFM

The HFM is helpful to select the right performance grading of an asphalt binder according to the climatic condition in the area of uses. In permafrost regions, asphalt pavement is preferred because its flexible properties are advantageously adapted to differential settlement that is caused by the warming of the underlying frozen soils^29^. Asphalt pavement is typically placed on the top of an embankment. It is critical to select the right performance grading of the asphalt binders to the right climate condition in the area of uses. Performance grading is a function of annual maximum and minimum pavement surface temperature, with one being the average seven-day maximum pavement temperature and the other being the minimum^30,31^. With the HFM, these temperature extremes can be precisely estimated according to the texture and color of the asphalt pavement and to the specific climatic conditions.

The HFM is powerful to estimate the heat gain and loss at the embankment surface precisely. The most concern heat flux component at the ground surface is the conductive heat flux, which mainly determines the temperature of the underlying frozen soils^32–34^. The HFM can reliably estimate the conductive heat flux to an embankment in a specific climate condition. Under the context of global warming, the HFM can be a useful tool to envision if the underlying permafrost can be cooled by varying embankment surface properties like albedo and emissivity. In addition to the conductive heat flux, how the radiant heat flux partitions at the embankment surface can be calculated. Understanding the heat flux balance at the ground surface is crucial to develop cool embankments and cool pavements for preserving the underlying permafrost under global warming.

The HFM is robust to find practical solutions to eliminate the thermal asymmetry of a roadway embankment in permafrost regions. After an embankment is built, the southern side slope absorbs more solar irradiance than the northern one does. As a result, the permafrost under the southern side slope is typically some Celsius degrees warmer than that under the northern one, a phenomenon that is termed as the sunny-shady effect and has been blamed as the culprit to the longitudinal cracks of the embankment^35^. Since this effect is caused by differential solar irradiance on both embankment side slopes, it can be mitigated by increasing the albedo of the southern side slope. But it remains unknown that for a specific embankment, increasing the albedo of the southern side slope to which degree can equalize the heat gain of both side slopes and thus eliminate the sunny-shady effect. The HFM can fill this knowledge gap and serves as a powerful tool to estimate the sunny-shady effect.

The HFM is indispensable to precisely calculate the deformation of warming permafrost under the embankment. Building embankment alters the thermal regimes such that the embankment absorbs more sunlight than the adjacent natural ground surface even if the surface materials are the same^36^. Plus the construction thermal disturbance, the permafrost underlain a roadway embankment has been found 0–1 °C warmer than the adjacent natural ground, especially in warm permafrost regions^37^. It has been observed that this temperature increment has caused 10–20 cm settlement to the embankment in warm permafrost region, and that the warming of permafrost still continues due to the global warming^38^. Correctly simulating the temperature increment caused by global warming and construction-induced warming is crucial to predict the embankment’s settlement, which is directly correlated to the damage of the embankment. As the HFM is far more reliable to predict the temperature of the underlying permafrost (Figs. 2 and 3), it is thus indispensable to foresee the serviceability of embankments in permafrost regions.

Other applications of the HFM may be vast but cannot be exhausted herein. For instance, the use of the HFM is not limited to the embankments in permafrost regions but also to those in seasonal frozen regions. It can be used to study on the influences of parameters like surface albedo, surface emissivity, surface roughness, pavement thermal inertia, and other controllable factors on the temperature of the underlying soils and of the pavement structures. It can be also adopted to estimate the impacts the uncontrollable factors like solar irradiance, wind speed, air temperature, and others on the soil temperature. Furthermore, it can be employed to estimate the temperature under embankments that is proactively cooled by inserting the embankment with thermosyphons, covering the side slope with crushed rock layer, and embedding the embankment with air convective duct, etc. While other applications of the HFM is unlimited and await further studies, this study is just a starting of the use of HFM to predict the temperature of permafrost under a roadway embankment.

## Conclusion

This study proposed a heat-flux model (HFM) for the heat transfer at the upper surface of an embankment in permafrost regions. Different from traditional temperature-controlled model (TCM) that considers the annual ground-surface temperature varied as a sinusoidal wave, the HFM jointly considers the solar irradiance, heat convection, and thermal irradiance on the ground surface, as well as the shading effect of the embankment on the adjacent ground. The HFM model thus can be used to predict the influence of ground-surface thermal properties and local weather on the temperature of the permafrost under an embankment. The permafrost temperature predicted by HFM is compared against that observed ones. It is found that the HFM model can predict the permafrost soils’ temperature precisely, for instance, with R^2^ = 0.975, a mean error of 0.134 °C and a standard deviation of 0.702 °C. In comparison, the TCM-predicted temperature is also compared against the observed one. It is found that TCM predicts the permafrost soil temperature in a lower accuracy.

Temperatures predicted by the HFM are 0.1–0.2 °C lower than those predicted by the TCM, but the temperature-varying trend and temperature contour are similarly. It is concluded that if the temperature trend of the soil under an embankment is of concern, the TCM can be recommended if the mean annual ground-surface temperature and its amplitude can be guessed precisely. If the sensitivity of the weather data and ground-surface thermal properties on the permafrost temperature is of concern, the HFM is recommended but the solar reflectance of the ground surface must be weighted wisely. Further studies are expected to understand the energy-based solar reflectance of the natural ground surface and of the embankment surface so that the solar absorption in the HFM is weighted precisely. The HFM can be applied to guide the selection of the performance grading of asphalt pavement in permafrost regions, to understand the heat partition on the embankment surface, to understand the sunny-shady effect of embankments, to calculate the deformation of embankment, and explore other applications related to the HFM.

## Supplementary Information

Supplementary Information.

## Data Availability

Some or all data, models, or code that support the findings of this study are available from the corresponding author upon reasonable request.

## English symbols

*T*: Temperature (°C)
*T*_*s*_: Ground-surface temperature (°C)
*T*_*a*_: Air temperature (°C)
*T*_*d*_: Dew point temperature (°C)
*T*_*e*_: Bottom limit temperature (°C)
*T*_*f*_: Upper limit temperature (°C)
*T*_*0*_: Mean annual temperature (°C)
*c*: Heat capacity (J/(kg·K))
*c*_*eq*_: Equilibrium heat capacity (J/(kg·K))
*t*: Time (s)
*A*: Amplitude of the temperature (°C)
*I*: Solar irradiance (W/m^2^)
*I*_*b*_: Beam irradiance (W/m^2^)
*I*_*bs*_: Beam irradiance on the side slope (W/m^2^)
*I*_*bg*_: Beam irradiance on the ground (W/m^2^)
*I*_*d*_: Diffuse irradiance (W/m^2^)
*I*_*ds*_: Diffuse irradiance on the side slope (W/m^2^)
*I*_*dg*_: Diffuse irradiance on the ground (W/m^2^)
*I*_*s*_: Solar irradiance on the side slope (W/m^2^)
*R*: Reflectance (–)
*L*: The net long-wave radiation (W/m^2^)
*L*_*d*_: Downward thermal irradiance (W/m^2^)
*L*_*u*_: Upward long-wave radiation (W/m^2^)
*H*: Heat convection (W/m^2^)
*E*: Evaporation (W/m^2^)
*G*: Conduction (W/m^2^)
*h*_*c*_: Heat convection coefficient (W/(m·°C))
*v*: Wind speed (m/s)
*z*: Height (m)
*N*: Day number of the year (–)
*k*_*y*_: Sky cleanness factor (–)
*h*: Height of the embankment (m)
*x*_0_: Width of the shaded area (m)
*F*_*x→sky*_: Sky view factor (–)
*w*: Water content (–)
*L*_*w*_: Latent heat of water (kJ/kg)

## Greek symbols

*ρ*: Density (kg/m^3^)
*λ*: Heat conductivity (W/(m⋅K))
*ε*: Thermal emissivity (–)
*σ*: Stefan–Boltzmann constant (–)
*χ*: Relative humidity (–)
*β*: Latitude (rad)
*δ*: Solar declination angle (rad)
*α*: Solar hour angle (rad)
*θ*: Solar zenith angle (rad)
*γ*: Solar azimuth angle (rad)
*γ*_*e*_: Orientation of the embankment (rad)
*η*: Angle of the side slope (rad)
*ψ*: Azimuth of the side slope (rad)
*ω*: Angular frequency (rad/s)
*ϕ*: Phases (rad)

## Subscripts

*us*: Unfrozen soil
*fs*: Frozen soil
*w*: Water
*u*: Unfrozen water
*i*: Ice
*y*: Sky

## Abbreviations

NG: Natural ground surface
S: Side slope
SS: South-facing side slope
NS: North-facing side slope
QTH: Qinghai-Tibet Highway
QTR: Qinghai-Tibet Railway
QTP: Qinghai-Tibet Plateau
