# Supplementary material for: A heat-flux upper boundary for modeling temperature of soils under an embankment in permafrost region
Source: Sci Rep. 2022 Aug 2;12:13295. doi: 10.1038/s41598-022-17529-w (PMC9345884; doi:10.1038/s41598-022-17529-w)
Supplement: Supplementary file 1 — Supplementary Information. [file 41598_2022_17529_MOESM1_ESM.pdf]

Title: A heat-flux upper boundary for modeling temperature of soils under an embankment in permafrost region

Authors: Tianyu Wang [*], Li-E Yan

Appendix A:

As the thermal conductivity of water is different from that of ice, the λ of a soil at frozen and unfrozen states can be estimated using a step-wise function:

$$\lambda = \begin{cases} \lambda_f, \; T < T_e \\ \lambda_f + \frac{\lambda_u - \lambda_f}{T_e - T_f}(T_e - T), \; T_e < T \le T_f \\ \lambda_u, \; T > T_f \end{cases} \tag{A1}$$

where λ (W/(m·K)) is the thermal conductivities and T (°C) is temperature, the subscript of "*u*" and "*f*" represent the unfrozen and frozen states, respectively; T_e is a temperature below which the phase change is negligible;

The apparent heat capacity of the soil, c_{eq}, is determined by:

$$c_{eq} = \begin{cases} \frac{C_{us} + w_u C_w}{1+w}, \; T > T_f \\ \frac{L_w}{1+w}\frac{\partial w_i}{\partial T} + \frac{C_{fs} + (w - w_u)C_i + w_u C_w}{1+w}, \; T_e < T \le T_f \\ \frac{C_{fs} + w_i C_i}{1+w}, \; T < T_e \end{cases} \tag{A2}$$

where the subscripts of *us*, *fs*, *w*, *u*, and *i* stand for unfrozen soil, frozen soil, water, unfrozen water, and ice, respectively; *w* is water content of the soil; L_w=344 kJ/kg is the latent heat of water. The unfrozen water content in frozen soil:

$$w_u = a(-T)^{-b} \tag{A3}$$

where a and b are two constants depending on the soils' properties [38]. The ice content of a frozen soil w_i is:

$$w_i = w - w_u \tag{A4}$$

According to Eq. (A3), T_f can be found from:

$$T_f = -\left(\frac{a}{w}\right)^{1/b} \tag{A5}$$

Neglecting phase change of unfrozen water when $dw_u/dT \le 0.001$, one has T_e

$$T_e = -\left(\frac{ab}{0.001}\right)^{\frac{1}{b+1}} \tag{A6}$$

Appendix B:

Daily mean temperature predicted by the HFM (Fig.B1 a) and the TCM (Fig.B1 b) as well as the temperature differences (Fig.B1 c) between the two models are shown. The data in Fig. B1 is on 19 October in the 10th year; and that in Fig. B2 is on 19 October in the 50th year. October 19 is the day when the thawing penetrating to the deepest. It can be found that the temperature pattern, temperature trend, and the difference of the two predictions are highly similar.

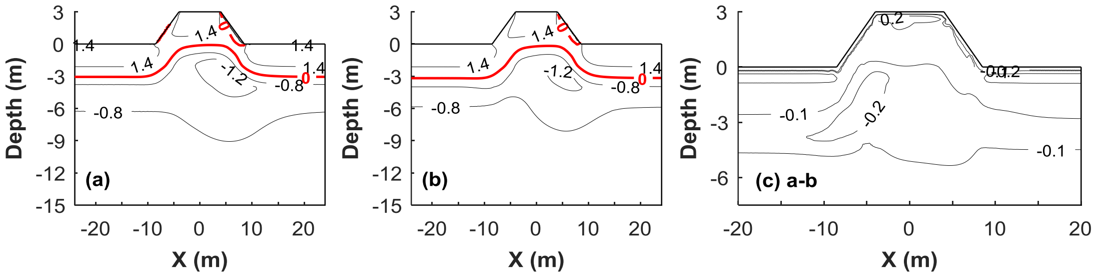

Fig.B1 Comparison between the HFM-predicted temperatures and TCM-predicted ones on October 19 in the 10th year after construction. (a) HFM-predicted temperature, (b) TCM-predicted temperature, and (c) the difference between HFM-predicted temperatures and TCM-predicted ones.

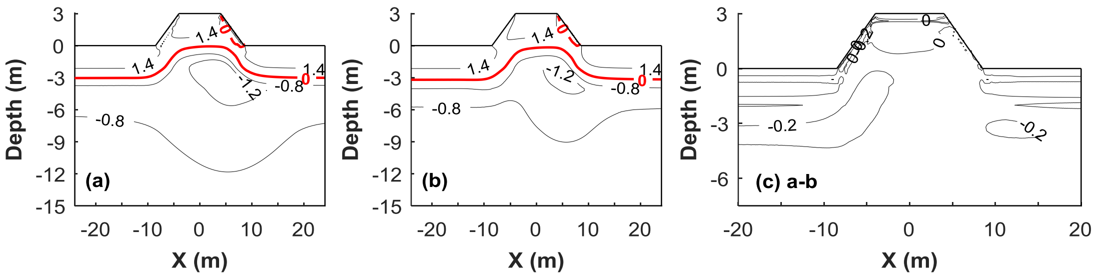

Fig.B2 Comparison between the HFM-predicted temperatures and TCM-predicted ones on October 19 in the 50th year after construction. (a) HFM-predicted temperature, (b) TCM-predicted temperature, and (c) the difference between HFM-predicted temperatures and TCM-predicted ones.
